# A New Magnetically Separable BaFe_2_O_4_ Acid Catalyst for Sustainable Biodiesel Production: L_9_ Taguchi Optimization and Robust Recyclability

**DOI:** 10.1021/acsomega.5c10408

**Published:** 2026-01-27

**Authors:** Matheus Arrais Gonçalves, Hiarla Cristina Lima dos Santos, Vicente da Silva Lima, Heverton Jonnys Feitosa da Silva, Deborah da Cunha Fonseca, Thaissa Saraiva Ribeiro, Beatriz dos Santos Silva, Alexandre da Cas Viegas, Leyvison Rafael Vieira da Conceição

**Affiliations:** † 37871 Federal University of Pará, Institute of Exact and Natural Sciences, Graduate in Chemistry Program, Laboratory of Catalysis and Oleochemical, Belém, Pará 66075−110, Brazil; ‡ 37871 Federal University of Pará, Institute of Biological Sciences, Graduate in Biotechnology Program, Belém, Pará 66075−110, Brazil; § 119528 Federal Institute of Education, Science and Technology of Pará, Graduate Program in Materials Engineering, Belém, Pará 66093−020, Brazil; ∥ 28117 Federal University of Rio Grande Do Sul, Institute of Physics, Porto Alegre, Rio Grande Do Sul 90035−190, Brazil

## Abstract

The MoO_3_/BaFe_2_O_4_ catalyst was synthesized via a combined coprecipitation and wet impregnation approach and subsequently applied in the methyl biodiesel production from waste cooking oil (WCO). The material was characterized using surface acidity measurements, XRD, FTIR, SEM, EDS, and VSM techniques. The results confirmed: (i) the successful synthesis of barium ferrite (BaFe_2_O_4_), and (ii) the effective impregnation of MoO_3_ onto the ferrite matrix. Process optimization was conducted using the Taguchi L_9_ methodology, evaluating four operational parameters: temperature (120–180 °C), methanol:WCO molar ratio (20:1–40:1), catalyst concentration (2–10 wt %), and reaction time (1–5 h). The high coefficient of determination (*R* ^2^ = 0.9410) confirmed the model’s robustness and predictive capability for ester content. The optimal conditions (temperature = 172 °C, methanol:WCO molar ratio = 28:1, catalyst concentration = 7.5 wt %, time = 4 h) yielded a maximum conversion of 96.4% into methyl esters. Notably, the catalyst exhibited: (i) exceptional recyclability, maintaining efficiency above 90% after seven consecutive cycles, and (ii) facile magnetic separation under an external magnetic field. Furthermore, the physicochemical properties of the produced biodiesel fully complied with ASTM D6751 and EN 14214 standards, underscoring the catalyst’s potential for industrial-scale transesterification processes.

## Graphical abstract

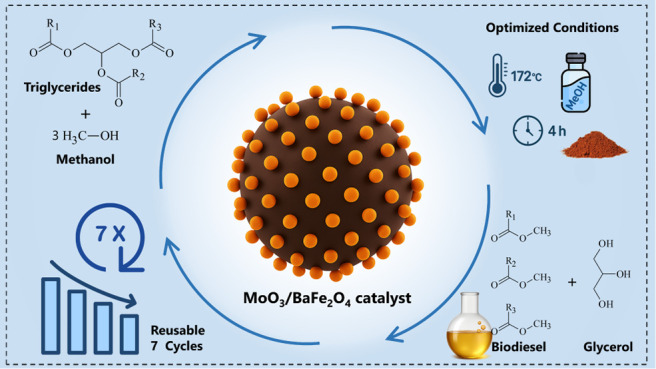

## 1 Introduction

Rising fuel prices, climate change, and environmental pollution have driven the search for cleaner energy alternatives to progressively replace the current fossil fuel-based energy matrix.^1,2^ Among emerging energy options, biodiesel shows promising potential compared to fossil fuels, notably reducing net emissions of carbon dioxide, carbon monoxide, and particulate matter by 7%, 46.7%, and 66.7%, respectively.^3,4^ Consequently, countries like Brazil are moving toward commercializing petroleum diesel blended with 20% biodiesel (B20 mixture) to offer a less polluting fuel.^5^

Biodiesel, defined as fatty acid methyl or ethyl esters, is synthesized through biomass conversion in the presence of alcohol and typically a catalyst to accelerate the process.^6,7^ Among studied feedstocks, residual biomasses such as animal fats, waste vegetable oils, and soapstocks are particularly noteworthy due to their low cost, which reduces the overall production expense.^8,9^

Among the main feedstocks, waste cooking oil (WCO) stands out due to its high availability, with approximately 16.5 million tons produced globally each year, primarily in countries such as the United States, China, and Brazil.^10,11^ In addition, low cost and environmental benefits are other key aspects that explain the widespread use of WCO, since reusing this waste not only mitigates the environmental pollution caused by improper disposal, but also adds value to a waste material, contributing to the principles of the circular economy. For example, it is estimated that 1 L of used cooking oil can contaminate up to 20,000 L of water if disposed of directly into the aquatic environment, in addition to causing blockages in sewage networks, increasing the cost of wastewater treatment, contaminating soil and water bodies, and damaging aquatic biodiversity.^8^

However, despite its promise, WCO contains a high percentage of free fatty acids (FFA), which hinders its use with basic catalysts due to the occurrence of undesirable side reactions, such as saponification.^12,13^ In this context, acid catalysts are more suitable for processing residual feedstocks, particularly heterogeneous catalysts, owing to advantages such as resistance to equipment corrosion, and the possibility of recovery and reuse in the reaction process, factors that contribute to reducing operational costs.^14,15^ Given these advantages, various solid acid catalysts have been extensively studied for biodiesel synthesis, including some materials such as zeolites, mesoporous, ferrites, coals etc.^16–20^

Magnetic solid catalysts have gained prominence and are increasingly studied due to their enabling simplified separation from the reaction medium through the application of a magnetic field, eliminating the need for more sophisticated and costly techniques such as centrifugation.^21–23^ In this context, various magnetic materials have been investigated as promising catalysts for biodiesel synthesis, especially when combined with other catalytically active oxides.^7,24–26^ Salimi et al.^27^ reported the use of barium ferrite (BaFe_2_O_4_) impregnated with potassium oxide (K_2_O), achieving 97.63% conversion to fatty acid methyl esters under optimized conditions: temperature of 65 °C, methanol:WCO molar ratio of 12:1, and catalyst concentration of 4 wt %. In the study by Figueiredo et al.,^28^ the authors investigated the application of MoO_3_ impregnated on zeolites (ZSM-5) and mesoporous silica (SBA-15) for biodiesel production and obtained 79.2% ester conversion under the following conditions: temperature of 150 °C, methanol:oil molar ratio of 20:1, catalyst concentration of 6 wt %, and reaction time of 4 h.

In relation to the active phase used in acid catalysis, tungsten oxide (WO_3_) and molybdenum oxide (MoO_3_) have been gaining prominence due to the versatility of their redox properties, since molybdenum has several oxidation states, such as Mo^6+^ (most stable), Mo^5+^, Mo^4+^, Mo^3+^, and Mo^2+^, it can be widely applied in industrial catalytic processes.^17–19^

Given this context, the present work proposes the development of a novel acid magnetic catalyst based on BaFe_2_O_4_/MoO_3_ for sustainable biodiesel production from waste cooking oil (WCO). This study is distinguished by (i) addressing an important scientific gap, as acid magnetic catalytic systems based on barium ferrite have not yet been reported for this application, and (ii) employing an optimized approach via the Taguchi L_9_ method to systematically evaluate key reaction parameters (temperature, methanol:WCO molar ratio, catalyst concentration, and time). Thus, it is worth emphasizing that the results obtained may contribute significantly to the advancement of more efficient and sustainable catalytic processes in biofuel production.

## 2 Experimental Procedure

### 2.1 Materials

For the synthesis of the ferrite and magnetic catalyst, the following reagents were used: barium nitrate (Ba­(NO_3_)_2_, Dinâmica, 99%), iron­(III) chloride (FeCl_3_, Merck, 98%), nitric acid (HNO_3_, Isofar, 99%), and ammonium heptamolybdate ((NH_4_)_6_Mo_7_O_24_·4H_2_O, Dinâmica). For the biodiesel synthesis, methanol (CH_3_OH, Dinâmica, 99.8%) was acquired from a commercial supplier, and waste cooking oil (WCO) was obtained from a local market in Belém, Pará, Brazil. For the catalyst recyclability study, hexane (C_6_H_14_, Exôdo Científica, 99%) and ethanol (CH_3_CH_2_OH, Dinâmica, 99.8%) were employed. Chromatographic analysis utilized methyl heptadecanoate (C17:1, Sigma-Aldrich, 99%) and heptane (C_7_H_16_, Dinâmica, 99.5%). Surface acidity of the catalyst was determined using sodium hydroxide (NaOH, Neon, 97%) and hydrochloric acid (HCl, Isofar, 37%). All chemical reagents and solvents were of analytical grade and used without further purification. The physicochemical properties of the WCO are presented in Table 1, and the determination methodology can be found in the Supporting Information.

**Table 1:** Physicochemical Properties of WCO to the Synthesized Biodiesel

Properties	Value
**Physicochemical properties**	
Acid value, (mg KOH g^–1^) (AOCS Cd 3d–63)	2.2
Saponification value, (mg KOH g^–1^) (AOCS Tl 1a–64)	186.3
Viscosity at 40 °C, (mm^2^ s^–1^) (ASTM D445)	36.3
Moisture content, (%) (AOCS Ca 2b–38)	0.1
Molecular weight, (g mol^–1^)	855

### 2.2 Preparation of Heterogeneous Acid Magnetic Catalyst (MoO_3_/BaFe_2_O_4_)

#### 2.2.1 Synthesis of Barium Ferrite (BaFe_2_O_4_)

The synthesis of barium ferrite was carried out using a coprecipitation method adapted from the procedure described by Salimi et al.,^27^ as outlined in Figure 1a. Initially, precursors of barium and iron were weighed in a molar ratio of Fe:Ba = 2:1 and dissolved in 100 mL of distilled water under constant mechanical stirring. The resulting mixture was acidified with concentrated HNO_3_ until reaching pH = 3 to ensure complete dissolution of the salts. The system was maintained under constant stirring during the slow addition of 4 mol L^–1^ NaOH solution until pH = 12 was reached to promote precipitate formation, and then kept at 80 °C for 4 h for particle aging. The precipitated material was vacuum-filtered and washed with distilled water until the filtrate reached neutrality (pH ≈ 7). The solid obtained was dried in an oven at 80 °C for 12 h and subsequently calcined in a muffle furnace at 900 °C for 2 h using a heating rate of 10 °C min^–1^ to obtain the final BaFe_2_O_4_ product.

**Figure 1:**
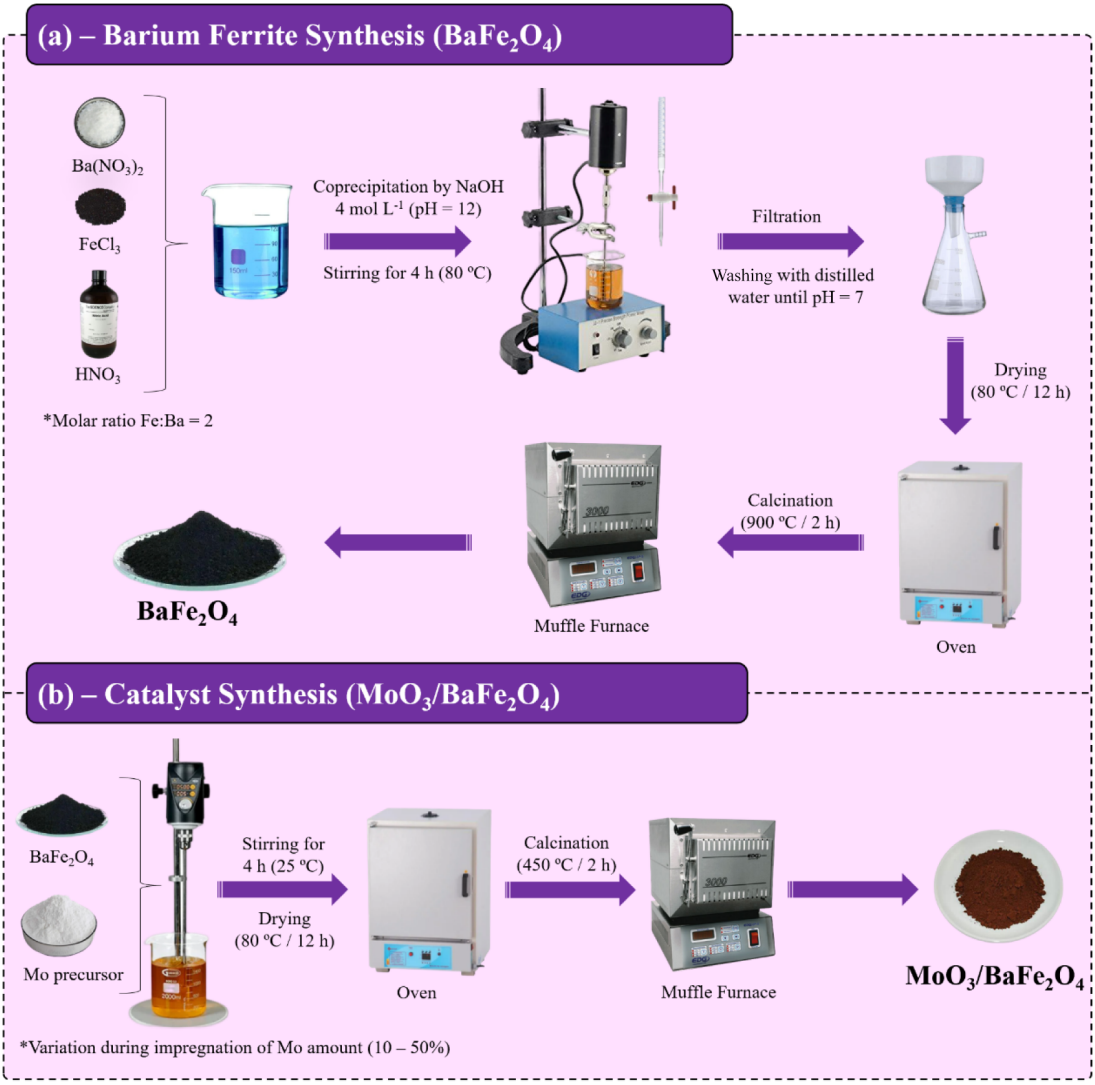
Schematic illustration of (a) BaFe_2_O_4_ and (b) MoO_3_/BaFe_2_O_4_ synthesis.

#### 2.2.2 Magnetic Heterogeneous Catalyst Synthesis (MoO_3_/BaFe_2_O_4_)

The heterogeneous acid catalyst MoO_3_/BaFe_2_O_4_ was prepared via wet impregnation following the methodology adapted from Santos et al.,^29^ as illustrated in Figure 1b. Initially, a predetermined amount of presynthesized BaFe_2_O_4_ was weighed and added to a beaker containing 40 mL of distilled water and (NH_4_)_6_Mo_7_O_24_·4H_2_O at different concentrations corresponding to 10, 20, 30, 40, and 50 wt % of Mo. The mixture was maintained under constant mechanical stirring at room temperature for 4 h. After this period, the material was subjected to drying in an oven at 80 °C for 12 h, followed by calcination in a muffle furnace at 450 °C for 2 h with a controlled heating rate of 10 °C min^–1^ to facilitate the transformation of the precursor into MoO_3_ and its fixation onto the ferrite structure. The obtained catalysts were designated as z-MoO_3_/BaFe_2_O_4_, where z represents the percentage content of molybdenum (10–50%) in the final catalytic material.

### 2.3 Materials Characterization Techniques

The acid sites responsible for the catalytic activity of the heterogeneous MoO_3_/BaFe_2_O_4_ catalyst were measured by acid–base titration according to a methodology adapted from Boehm (1994). Initially, 0.25 g of the catalyst was placed in 30 mL of a standardized 0.1 mol L^–1^ HCl solution and stirred for 4 h at room temperature. Subsequently, the sample was separated using an external magnetic field, and the supernatant was mixed with 15 mL of a standardized 0.1 mol L^–1^ NaOH solution. This mixture was then titrated with a 0.1 mol L^–1^ HCl solution in the presence of phenolphthalein as an indicator.

The crystallographic structure of MoO_3_, BaFe_2_O_4_, and MoO_3_/BaFe_2_O_4_ was evaluated by X-ray diffraction using a Bruker D2 PHASER diffractometer under the following operational conditions: Cu radiation (Kα = 1.54 Å), 40 kV, 30 mA, and a 2θ range of 10° to 90°. The chemical functional groups present in MoO_3_, BaFe_2_O_4_, and MoO_3_/BaFe_2_O_4_ were detected by FTIR spectroscopy using a Shimadzu Prestige 21 spectrometer. The spectral analysis range was 2000–400 cm^–1^, with a resolution of 4 cm^–1^ and 32 scans.

The surface morphological properties of MoO_3_, BaFe_2_O_4_, and MoO_3_/BaFe_2_O_4_ were investigated using a TESCAN VEGA 3LMU microscope. The surface chemical composition and elemental mapping of BaFe_2_O_4_, and MoO_3_/BaFe_2_O_4_ were obtained by EDS using an Oxford AZTec Energy X-Act microanalysis system with a resolution of 129 eV. The magnetic properties of BaFe_2_O_4_, and MoO_3_/BaFe_2_O_4_ were evaluated through hysteresis curves obtained using a Microsense EZ9 magnetometer at room temperature, with an applied magnetic field ranging from −20 kOe to 20 kOe.

### 2.4 Biodiesel Synthesis

The synthesized MoO_3_/BaFe_2_O_4_ catalyst was applied in biodiesel production via transesterification of WCO with methanol. Catalytic tests were conducted in a Parr Series 5000 Reactor under constant agitation at 900 rpm. The influence of molybdenum content (10, 20, 30, 40, and 50 wt %) in the final catalyst composition on biodiesel production was evaluated, along with other key reaction parameters. After each reaction, the products were magnetically separated from the catalyst and washed with 400 mL of distilled water at 80 °C to remove impurities. Following washing, the biodiesel samples were dried in an oven at 60 °C for 24 h and stored for subsequent determination of the ester content (*Y*_EC_, %) by gas chromatography.

### 2.5 Taguchi Orthogonal Design

The method developed by Genichi Taguchi enables the determination of optimal biodiesel production conditions using minimal required data, unlike other experimental designs that demand greater time and resources.^30–32^ In this study, an L_9_ orthogonal array was applied, comprising three levels for four independent variables, allowing process optimization through only nine experimental runs. The investigated variables were: temperature (*X*_1_, 120–180 °C), methanol:WCO molar ratio (*X*_2_, 20:1–40:1), catalyst concentration (*X*_3_, 2–10 wt %), and reaction time (*X*_4_, 1–5 h). The response variable selected for optimization was the ester content (*Y*_EC_, %) of the produced biodiesel.

Experimental results were analyzed using the signal-to-noise ratio (SNR), which evaluates the effect of independent variables on the desired response, with the Minitab 18 software. The SNR is a statistical parameter that quantifies the difference between the observed value and the desired target value, where the “signal” represents the desirable response and the “noise” corresponds to undesirable variations. The interpretation of the SNR value depends on the characteristic of the analyzed response, classified into three categories: *Larger-the-better* (LTB, higher is better), *Smaller-the-better* (STB, lower is better), and *Nominal-the-best* (NTB, nominal value is ideal), applied to maximize, minimize, or normalize the response variable, respectively. In this work, the LTB criterion was adopted for optimizing biodiesel ester content, aiming for maximum process conversion. SNR values were calculated according to eqs 1 and eq 2 :

1 $$\mathrm{Larger the better}\left(\mathrm{LTB}\right)=-10\log\frac{1}{n}\left(\overset{n}{\underset{j=1}{\sum }}\frac{1}{y_{j}^{2}}\right)$$

where:

2 $$y_{i}=\frac{1}{n}\left(\overset{n}{\underset{j=1}{\sum }}y_{ij}\right)$$

where *y*_*i*_ is the ester content value, *s*_*i*_^2^ is the variance, *n* is the number of experimental trials, *i* is the experimental number, and *j* is the trial number.

In addition to the SNR analysis, which alone does not precisely determine the individual effect and contribution of each variable in the process, an analysis of variance (ANOVA) was performed to statistically quantify the influence of each investigated parameter. This complementary statistical approach was essential to identify which factors exhibited statistical significance on the ester content (*Y*_EC_). The percentage contributions of each variable (*X*_1_: temperature, *X*_2_: methanol:WCO molar ratio, *X*_3_: catalyst concentration, and *X*_4_: reaction time) were calculated based on eqs eq 6, eq 6, and eq 6, allowing a quantitative assessment of the relative impact of each parameter on the biodiesel ester content. The combination of these analyses (SNR + ANOVA) provided a robust optimization of the process, identifying not only the optimal conditions but also the relative importance of each factor in the reaction system.

3 $$\mathrm{Contribution factor}\left(\%\right)=\frac{{\mathrm{SS}}_{f}}{{\mathrm{SS}}_{T}}$$

4 $${\mathrm{SS}}_{f}=\overset{3}{\underset{j=1}{\sum }}n{[{{(\mathrm{SNR}}_{L})}_{fj}-SNR_{T}]}^{2}$$

5 $$SS_{T}=\overset{9}{\underset{i=1}{\sum }}{[SNR_{i}-SNR_{T}]}^{2}$$

where SS_f_ and SS_T_ are the sum of squares of the factors and the total sum of squares of all variables, respectively. Additionally, SNR_L_ represents the average SNR value, and *n* denotes the number of runs for factor *f*.

### 2.6 Determination of Biodiesel Properties

The biodiesel samples obtained from catalytic tests were analyzed for ester content using gas chromatography (GC) following a methodology adapted from the European standard EN 14103, as described by Santos et al.^24^ A Varian CP-3800 gas chromatograph equipped with a flame ionization detector (FID) and a CP WAX 52 CB capillary column (30.0 m length, 0.32 mm diameter, and 0.25 μm film thickness) was employed. Helium was used as the mobile phase at a flow rate of 1.0 mL min^–1^, with an initial oven temperature of 170.0 °C and a heating rate of 10.0 °C min^–1^ to 250.0 °C (the FID and injector were maintained at the same temperature). Heptane served as the solvent, methyl heptadecanoate as the internal standard, and the injection volume was 1.0 μL. The ester content (*Y*_EC_) was calculated according to eq eq 6:

6 $$Y_{EC}\left(\%\right)=\frac{\left(\sum A_{T}\right)-A_{\mathrm{IS}}}{A_{\mathrm{IS}}}\times \frac{C_{\mathrm{IS}}}{C_{B100}}\times 100$$

where: ∑*A*_T_ is the sum of the total peak areas; *A*_IS_ is the peak area of the internal standard; *C*_IS_ is the concentration of the internal standard solution (mg L^–1^); *C*_B100_ is the concentration of the biodiesel sample after dilution (mg L^–1^).

Besides, The quality of the synthesized biodiesel was evaluated through key physicochemical properties following standardized American Society for Testing and Materials (ASTM) methods. Kinematic viscosity at 40 °C was determined according to ASTM D445 using a Cannon-Fenske viscometer (SCHOTT GERÄTE, 520 23). Density measurements at 20 °C were performed following ASTM D6890 with a KEM DAS-500 automatic densimeter. Acid value was quantified according to ASTM D664 methodology. Cold filter plugging point was measured using a TANAKA AFP-102 apparatus following ASTM D6371. Flash point determination was conducted according to ASTM D93 employing a TANAKA APM 7 automatic Pensky-Martens apparatus. Copper strip corrosion was evaluated following ASTM D130 specifications using a Koehler corrosion bath.

### 2.7 Catalyst Recyclability Study

After each biodiesel synthesis cycle, the MoO_3_/BaFe_2_O_4_ catalyst was recovered by applying an external magnetic field and washed four times with 30.0 mL of hexane and 40.0 mL of ethanol to ensure effective removal of impurities and reaction byproducts adsorbed on the catalyst surface. Subsequently, the catalyst was dried in an oven at 80 °C for 12 h to allow complete evaporation of residual solvents and reused in subsequent reaction cycles, enabling a systematic evaluation of its stability and catalytic performance over multiple reuses.

## 3 Results and Discussion

### 3.1 Influence of Molybdenum Amount on Catalyst Composition

The influence of the active phase on the final composition of the MoO_3_/BaFe_2_O_4_ catalyst was studied at molybdenum concentrations of 10.0, 20.0, 30.0, 40.0, and 50.0 wt %, relative to the ester content of the synthesized biodiesels, as shown in Figure 2.

**Figure 2:**
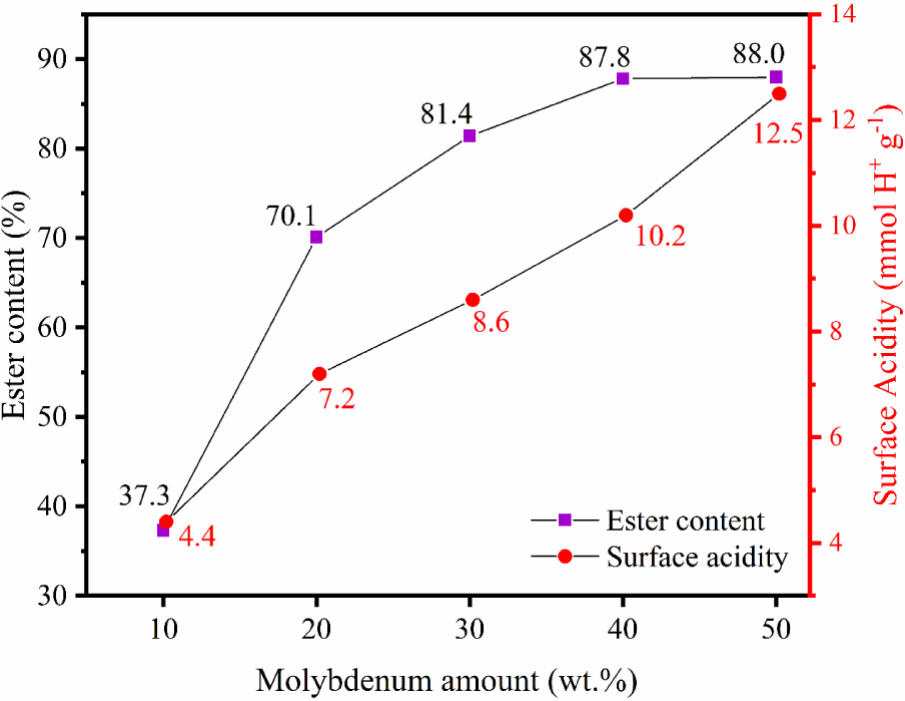
Influence of molybdenum amount on ester content (reaction conditions: reaction temperature of 150 °C, molar ratio methanol:WCO of 30:1, catalyst amount of 6 wt % and reaction time of 3 h).

Analysis of Figure 2 reveals that the highest ester conversion value (88.0%) was achieved using the catalyst with 50.0 wt % Mo, followed by the catalyst with 40.0 wt % Mo, which yielded 87.8% ester content. The ester content increased with higher Mo loadings, as evidenced by the values of 37.3%, 70.1%, and 81.4% for catalysts with 10.0, 20.0, and 30.0 wt % Mo, respectively. This trend is attributed to the increase in surface acid sites, which facilitate ion exchange during the reaction process.^12,33^

Furthermore, the surface acidity of the catalysts was analyzed, revealing a directly proportional relationship between Mo concentration and surface acidity values (Figure 2). The progressive increase in ester conversion with higher MoO_3_ loading can be attributed to the generation of new Lewis acid sites associated with coordinatively unsaturated Mo^6+^ species, since the strong electron-withdrawing character of Mo^6+^ enhances the polarization of Mo–O–Fe bonds, thereby increasing the overall surface acidity and facilitating carbonyl activation during transesterification.^14^ The surface acidity of the BaFe_2_O_4_ support was determined to be 1.2 mmol H^+^ g^–1^, indicating that the catalyst’s acidity primarily originates from the Lewis acid sites of the impregnated molybdenum.^12,28^ Based on these results, the 40-MoO_3_/BaFe_2_O_4_ catalyst was selected for further study. Although it provided a slightly lower ester conversion (87.8%) compared to the 50 wt % Mo catalyst (88.0%), it achieved a similar performance while using 10.0% less active phase, highlighting its efficiency and cost-effectiveness.

### 3.2 Catalyst Characterization of the Catalyst 40-MoO_3_/BaFe_2_O_4_

Figure 3 shows the X-ray diffractograms for MoO_3_, BaFe_2_O_4_, and the 40-MoO_3_/BaFe_2_O_4_ catalyst. The diffractogram corresponding to MoO_3_ (Green line) exhibits sharp and well-defined peaks, suggesting a highly crystalline structure characteristic of hexagonal (h-MoO_3_) and orthorhombic (α-MoO_3_) phases. The main diffraction peaks for MoO_3_ were detected at 2θ = 13.2°, 23.7°, 26.1°, 27.7°, 34.2°, 39.4°, 46.3°, 49.7°, 53.3°, 55.7°, 56.9°, 58.2°, and 59.3°. The diffractogram of the BaFe_2_O_4_ support (Purple line) displays peaks at 2θ = 19.3°, 28.7°, 30.7°, 32.5°, 33.2°, 34.5°, 37.4°, 40.6°, 42.9°, 44.6°, 55.4°, 56.9°, and 63.4°, which are consistent with its cubic spinel ferrite structure.^34,35^

**Figure 3:**
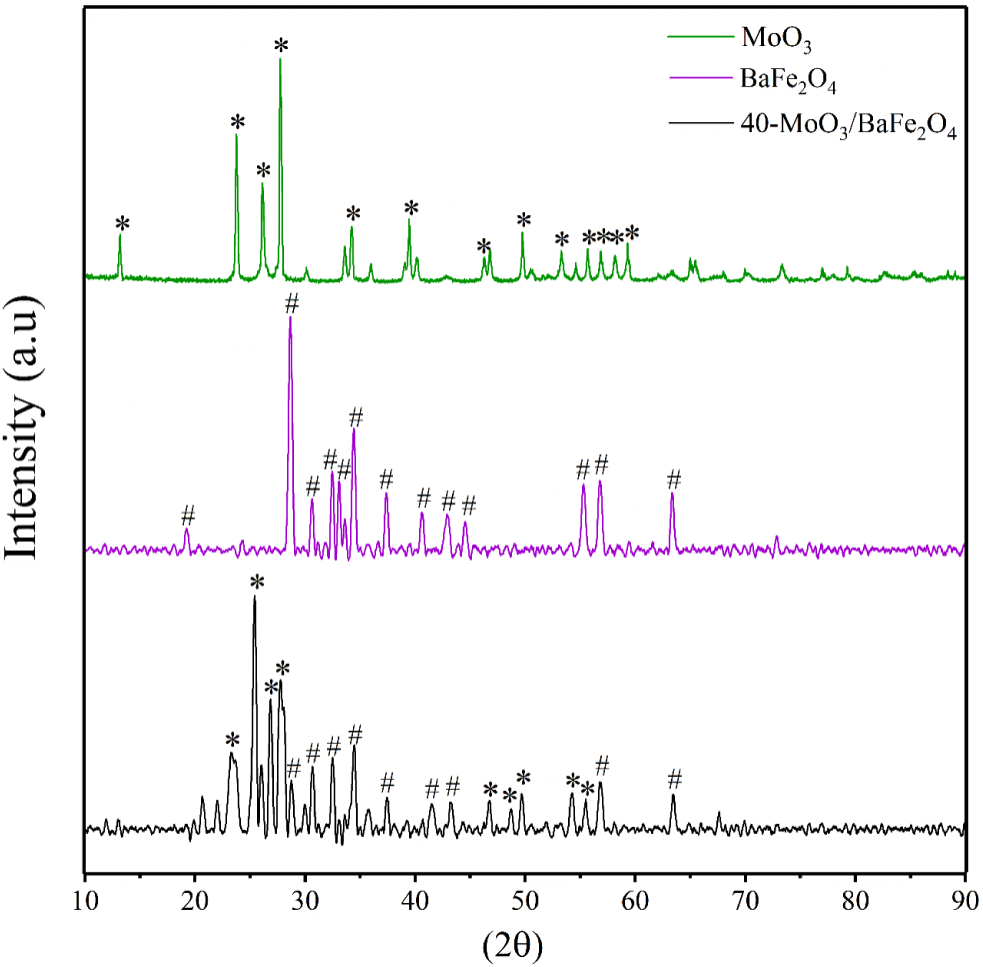
X-ray diffraction pattern for MoO_3_, BaFe_2_O_4_ and catalyst 40-MoO_3_/BaFe_2_O_4_.

For the diffractogram of the magnetic catalyst 40-MoO_3_/BaFe_2_O_4_ (Black line), the characteristic diffraction pattern of the magnetic support BaFe_2_O_4_ was identified at 2θ = 28.7°, 30.6°, 32.5°, 34.5°, 37.4°, 41.5°, 43.2°, 56.7°, and 63.5°, along with diffraction peaks corresponding to MoO_3_ at 2θ = 23.3°, 25.4°, 26.9°, 27.8°, 46.8°, 48.7°, 49.7°, 54.2°, and 55.5°.^7,14^

The functional groups present in MoO_3_, BaFe_2_O_4_, and the 40-MoO_3_/BaFe_2_O_4_ catalyst were evaluated by FTIR analysis, as shown in Figure 4. In the MoO_3_ spectrum (Green line), the main characteristic absorption bands were assigned to stretching vibrations of Mo–O bonds at 812 cm^–1^, O–Mo–O bonds at 842 cm^–1^, and Mo=O bonds at 983 cm^–1^.^36,37^ The spectrum of BaFe_2_O_4_ (Purple line) exhibits principal vibrational bands at 460 cm^–1^, 495 cm^–1^, 607 cm^–1^, and 769 cm^–1^, attributed to stretching vibrations of Ba–O and Fe–O bonds in octahedral and tetrahedral sites, while the band at 1423 cm^–1^ corresponds to O–Ba–O bonds in the spinel crystalline structure of the ferrite.^38,39^ For the 40-MoO_3_/BaFe_2_O_4_ catalyst (Black line), the spectrum shows characteristic absorption bands of BaFe_2_O_4_ at 541 cm^–1^ and 663 cm^–1^, assigned to Ba–O bond stretching vibrations in octahedral and tetrahedral sites of the spinel-type structure, respectively, along with bands around 808 cm^–1^, 921 cm^–1^, and 991 cm^–1^ characteristic of molybdenum–oxygen bonds.^7,14^

**Figure 4:**
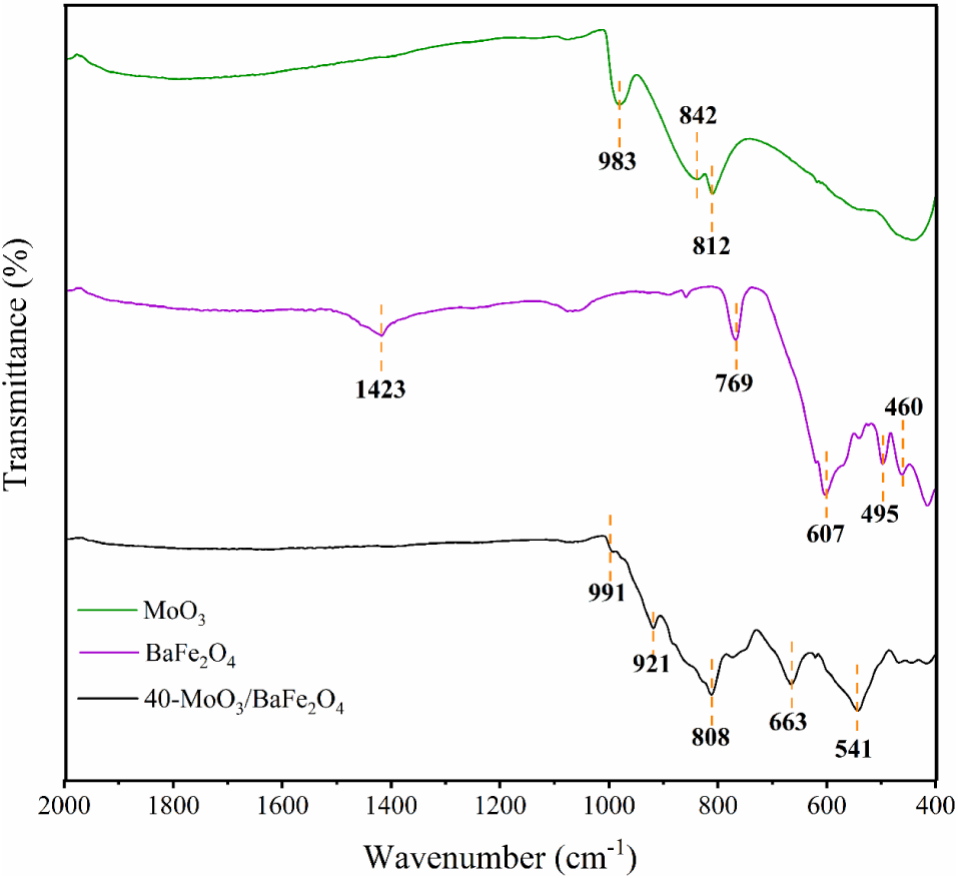
FTIR analysis of MoO_3_, BaFe_2_O_4_ and catalyst 40-MoO_3_/BaFe_2_O_4_.

A comparative analysis of the FTIR spectra for the 40-MoO_3_/BaFe_2_O_4_ catalyst reveals marked changes compared to the pure components. The intense band at 983 cm^–1^, attributed to the terminal Mo=O stretching vibration in pure, crystalline MoO_3_, is replaced by a low-intensity band at ∼991 cm^–1^ in the composite material. This blue-shift (shift to a higher wavenumber) indicates a strengthening of the Mo=O bond, consistent with the formation of under-coordinated and highly dispersed molybdenum species on the BaFe_2_O_4_ surface. The drastic reduction in band intensity confirms this high dispersion, signaling the presence of a reduced population of Mo=O sites that are effectively isolated and anchored to the magnetic support. These spectroscopic evidence corroborate the successful creation of an interface between the acidic active phase and the support, which is crucial for the catalytic performance.

The morphologies of MoO_3_, BaFe_2_O_4_, and the 40-MoO_3_/BaFe_2_O_4_ catalyst were investigated by SEM analysis. The micrographs obtained at resolutions of 1kx, 5kx, 10kx, and 15kx are illustrated in Figure 5. Figure 5a–d shows that the morphology of the MoO_3_ used consists of particle agglomerates with a tendency to form thin lamellae or elongated platelets.^33,40^ In the study by Sahoo et al.,^49^ which analyzed different MoO_3_ structures (orthorhombic, monoclinic, and hexagonal), the presence of hexagonally shaped rods was observed for synthesis temperatures below 400 °C, while temperatures above 400 °C resulted in deformation of the rod or lamella structure, characteristic of the stable orthorhombic phase.

**Figure 5:**
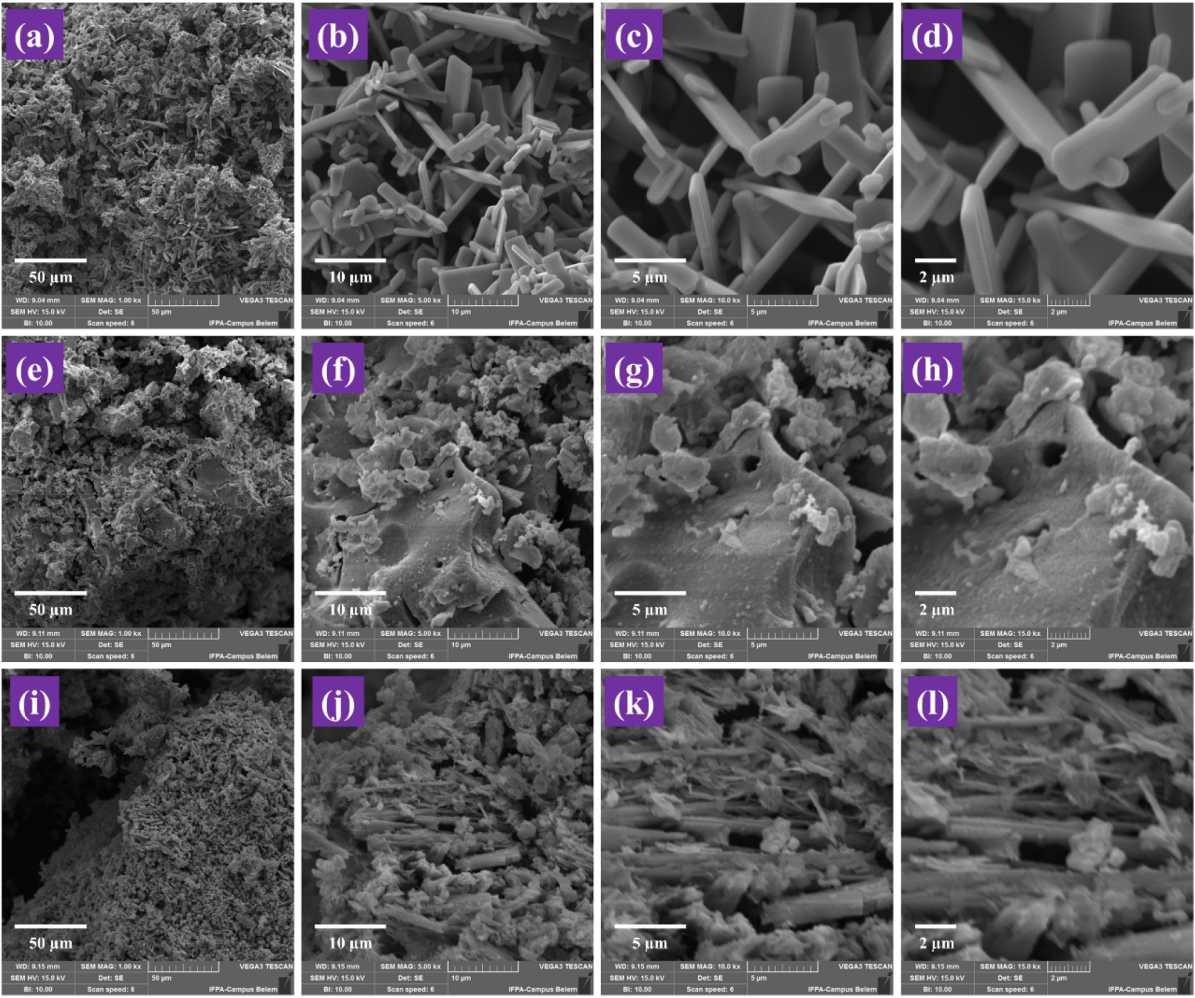
SEM images of (a) MoO_3_ 1kx (b) MoO_3_ 5kx, (c) MoO_3_ 10kx, (d) MoO_3_ 15kx, (e) BaFe_2_O_4_ 1kx (f) BaFe_2_O_4_ 5kx, (g) BaFe_2_O_4_ 10kx, (h) BaFe_2_O_4_ 15kx, (i) 40-MoO_3_/BaFe_2_O_4_ 1kx (j) 40-MoO_3_/BaFe_2_O_4_ 5kx, (k) 40-MoO_3_/BaFe_2_O_4_ 10kx and (l) 40-MoO_3_/BaFe_2_O_4_ 15kx.

Regarding the magnetic support BaFe_2_O_4_ (Figure 5e–h), no defined morphology is observable.^38,39^ Furthermore, the morphology of the 40-MoO_3_/BaFe_2_O_4_ catalyst (Figure 5i–l) exhibits rod-shaped particles on its surface due to the dispersion of MoO_3_ on BaFe_2_O_4_.

The EDS spectrum of 40-MoO_3_/BaFe_2_O_4_ (Figure 6a) revealed the presence of elements such as molybdenum (41.75%), oxygen (32.10%), barium (16.16%), and iron (8.99%). The Mo concentration on the catalyst surface (41.75%) is close to the theoretically estimated value (40.0%) for the active phase impregnation process on the catalytic support, demonstrating the success of the synthesis. Figure 6b shows the elemental mapping of the 40-MoO_3_/BaFe_2_O_4_ catalyst, along with individual element distributions, revealing a homogeneous dispersion of all elements on the support surface in the analyzed region.

**Figure 6:**
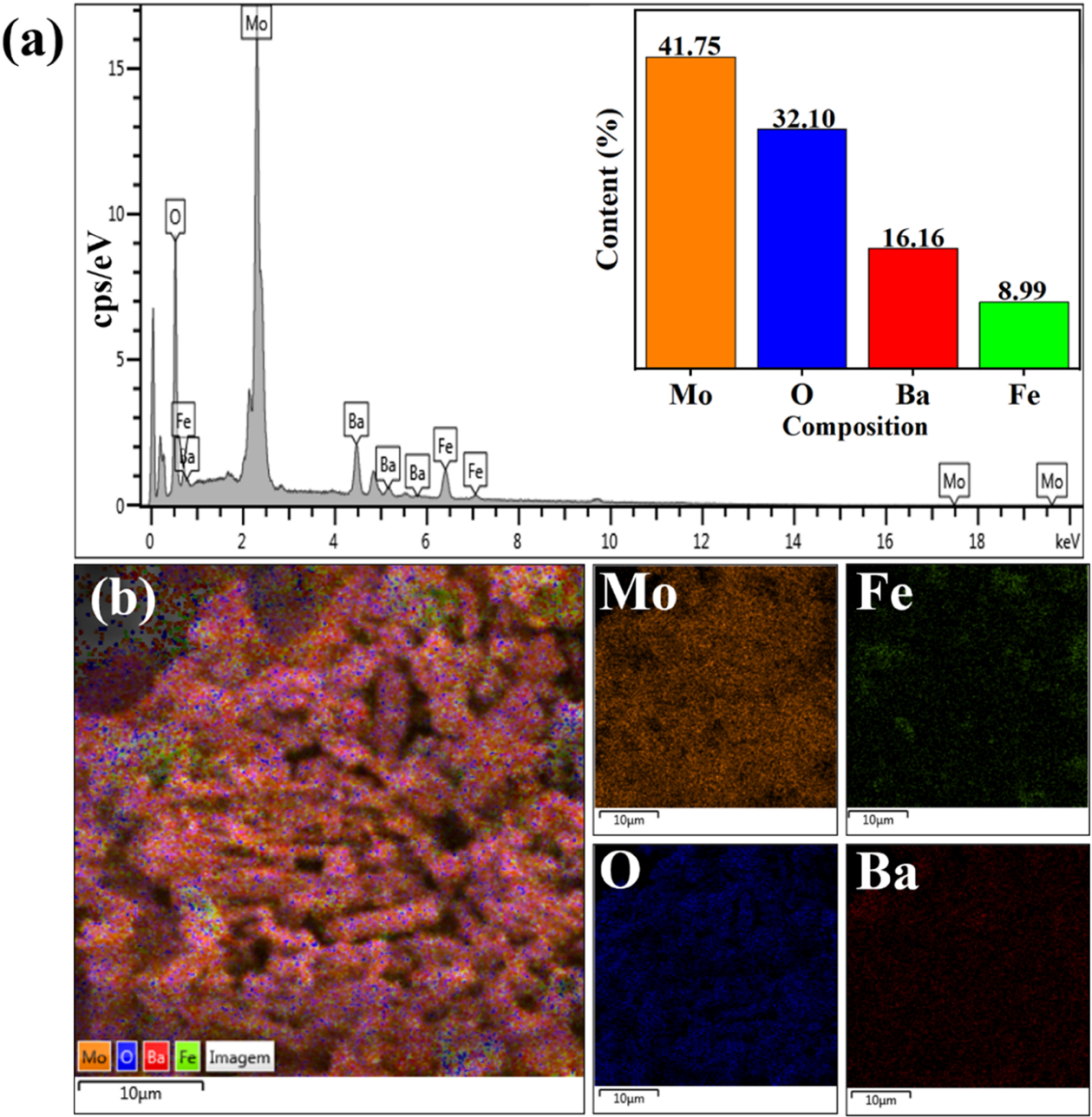
EDS composition of (a) catalyst 40-MoO_3_/BaFe_2_O_4_ and (b) maps of each component of the catalyst 40-MoO_3_/BaFe_2_O_4_.

The magnetic properties of BaFe_2_O_4_ and 40-MoO_3_/BaFe_2_O_4_, including saturation magnetization (Ms), remanent magnetization (Mr), and coercivity (Hc), were studied using vibrating sample magnetometry (VSM) through magnetization hysteresis curves (M) versus applied magnetic field (H), as shown in Figure 7.

**Figure 7:**
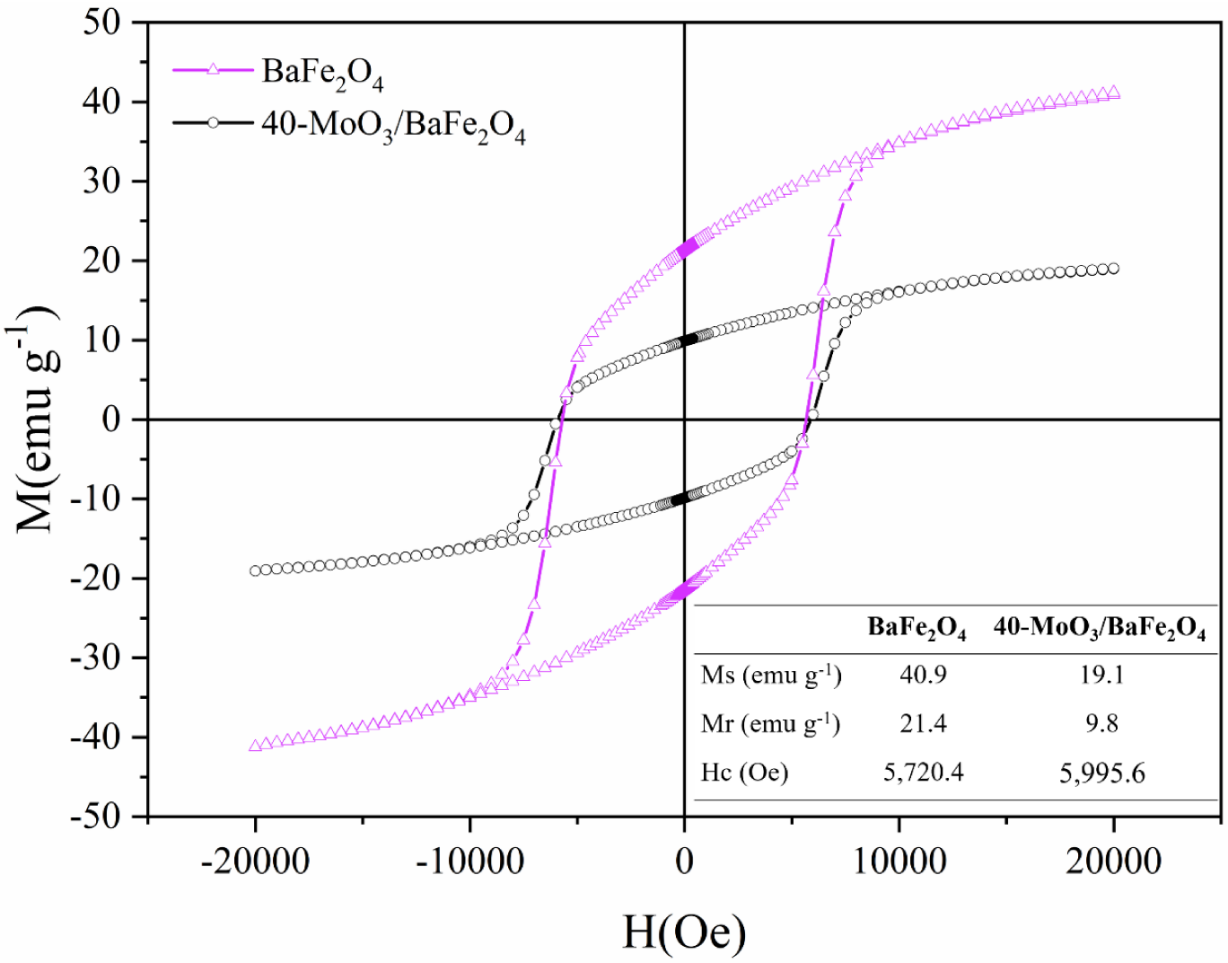
VSM analysis of BaFe_2_O_4_ and 40-MoO_3_/BaFe_2_O_4_.

The Ms values obtained were 40.9 emu g^–1^ for BaFe_2_O_4_ and 19.2 emu g^–1^ for 40-MoO_3_/BaFe_2_O_4_. The decrease in Ms from the support to the catalyst is attributed to the presence of 40% molybdenum (a nonmagnetic material) in the catalyst composition, along with the thermal treatment applied during the transformation of ammonium heptamolybdate into MoO_3_, which may have altered spin orientations and reduced the overall magnetic moment of the material. Nevertheless, the Ms value of the catalyst is sufficient for magnetic separation of 40-MoO_3_/BaFe_2_O_4_ using an external magnetic field and is significantly higher than those reported for other magnetic catalysts applied in biodiesel production. In the study by Santos et al.,^41^ an Ms value of 6.51 emu g^–1^ was obtained for a catalyst composed of manganese ferrite impregnated with an acidic oxide for biodiesel production. In another study by Abazari et al.,^42^ the NiFe_2_O_4_@SiO_2_/MgO catalyst, applied in biodiesel synthesis, exhibited an Ms of 30.8 emu g^–1^. Additionally, Wang et al.^43^ reported an Ms value of 3.7 emu g^–1^ for the magnetic catalyst ZrFe-SA-SO_3_ used in oleic acid esterification.

Moreover, the magnetization curves revealed distinct hysteresis loops for both BaFe_2_O_4_ and 40-MoO_3_/BaFe_2_O_4_, characterizing this ferrite as a hard magnetic material with ferrimagnetic behavior. This is evidenced by the presence of remanent magnetization (Mr) values of 21.4 emu g^–1^ for the ferrite and 9.8 emu g^–1^ for the catalyst when the applied magnetic field (H) is zero, along with significant coercivity (Hc) values of 5720.4 Oe for the ferrite and 5995.6 Oe for the catalyst, indicating high resistance to demagnetization.^14^

### 3.3 Taguchi Method for Process Optimization

The Taguchi L_9_ methodology was employed to optimize the biodiesel synthesis process through nine randomly conducted experiments, aiming to investigate and optimize the performance of the 40-MoO_3_/BaFe_2_O_4_ catalyst. The obtained *Y*_EC_ and SNR values are summarized in Table 2.

**Table 2:** Ester Content^a^

			**Level of factors**		
**Independent variables**	**Units**	**Codings**	**1**	**2**	**3**
Temperature	°C	*X*_1_	120	150	180
Molar ratio	mol/mol	*X*_2_	20	30	40
Catalyst dosage	wt %	*X*_3_	2	6	10
Reaction time	h	*X*_4_	1	3	5

**Runs**	**Order of runs**	***X***_**1**_	***X***_**2**_	***X***_**3**_	***X***_**4**_	***Y***_**EC**_ **(%)**	**SNR**
1	2	1	1	1	1	41.3	32.3
2	9	1	2	2	2	62.8	35.9
3	3	1	3	3	3	77.2	37.7
4	5	2	1	2	3	86.5	38.7
5	1	2	2	3	1	85.1	38.6
6	7	2	3	1	2	84.8	38.5
7	6	3	1	3	2	95.5	39.6
8	4	3	2	1	3	94.9	39.5
9	8	3	3	2	1	91.6	39.2
SNR_T_ - Overall mean							38.6

a SNR and SNR_T_ values for the L_9_ Taguchi matrix.

Analysis of Table 2 reveals that *Y*_EC_ values ranged from 41.3% to 95.5%, while SNR values remained within the interval of 32.3 to 39.6. Furthermore, to evaluate the impact of each variable on the process, the signal-to-noise ratio level (SNR_L_), defined as the algebraic mean of all SNR values for a specific level of a given variable, and the DSNR_L_, calculated as the difference between the maximum and minimum SNR_L_ values for each variable, were determined as illustrated in Table 3.

**Table 3:** SNR_L_ at Each Level of Factors and Rank of Factors

	SNR corresponding to the biodiesel ester content			
Level	*X*_1_	*X*_2_	*X*_3_	*X*_4_
1	35.3	36.9	36.8	36.7
2	38.6	38.0	37.9	38.0
3	39.5	38.5	38.6	38.7
DSNR_L_	4.1	1.6	1.8	1.9
Rank	1	4	3	2

The DSNR_L_ values were ranked from 1 to 4 according to their impact on the process, with 1 indicating the highest impact and 4 the lowest. The ranking revealed that temperature (*X*_1_) had the most significant influence on the biodiesel ester content, with a DSNR_L_ value of 4.1, followed by reaction time (*X*_4_) with DSNR_L_ = 1.9, catalyst concentration (*X*_3_) with DSNR_L_ = 1.8, and methanol:WCO molar ratio (*X*_2_) with DSNR_L_ = 1.6. The effect of each variable at three different levels on the biodiesel ester content in relation to SNR_L_ is shown in Figure 8. The SNR_L_ value increases as the variable becomes more influential in the process.

**Figure 8:**
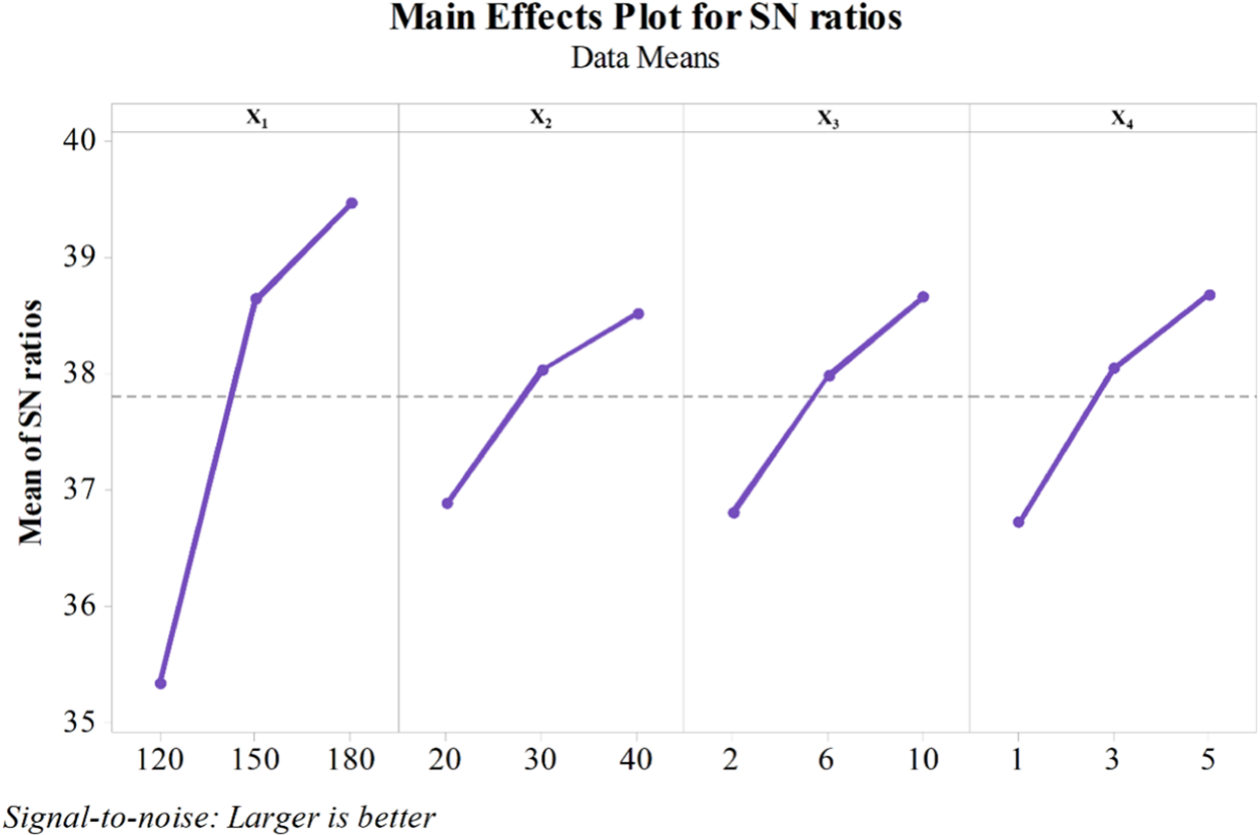
SNR_L_ of each factor at three different levels.

Furthermore, to quantify the influence of each variable and determine their percentage contribution to the ester content response, analysis of variance (ANOVA) was performed using eq eq 6. Analysis of Table 4 shows that the most predominant variable in the process is temperature (*X*_1_) with a percentage contribution of 73.4%, followed by reaction time (*X*_4_) with 11.2%, catalyst concentration (*X*_3_) with 9.1%, and methanol:WCO molar ratio (*X*_2_) with 6.3%.

**Table 4:** Findings of ANOVA for SNRs Coming from the Analysis^a^

Factors	DF	SS	MS	Percentage contribution (%)
*X*_1_	2	1826.2	913.1	73.4
*X*_2_	2	157.2	78.6	6.3
*X*_3_	2	226.2	113.1	9.1
*X*_4_	2	279.8	139.9	11.2
Error	0	-	-	-
Total	8	2489.5	-	100

a DF: Degree of freedom; SS: Sum of squares; MS: Mean square.

Based on the ANOVA results, a regression model was developed with a 95% confidence interval to describe the behavior of ester content in relation to the independent variables, as shown in eq 7

7 $$Y_{EC}\left(\%\right)=-38.5+0,56X_{1}+0.50X_{2}+1.53X_{3}+3.38X_{4}$$

Analysis of eq 7 allows verification of the behavior of the *Y*_EC_ values, both predicted and observed, as well as the residuals. Figure 9a shows the plot of predicted versus observed values, revealing an *R* ^2^ value of 0.9410, indicating that the developed model can explain approximately 94% of the *Y*_EC_ values in the process. Furthermore, based on the analysis of Figure 9b,c, it is observed that the errors are linearly distributed and not significant for the model, respectively, and that the residual distribution is random, thus indicating no violation of independence among the variables in the proposed model.^12^

**Figure 9:**
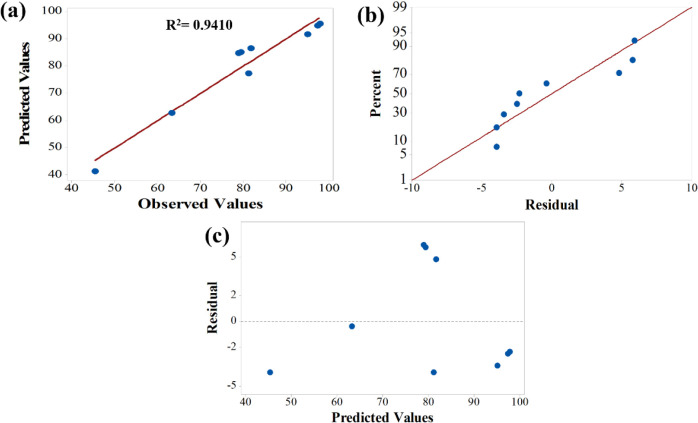
Plots of (a) Predicted vs. observed values for ester content, (b) Normal probability plots of the residuals and (c) Raw residuals vs. predicted values.

Figure 10 shows the contour plots for the variables temperature (*X*_1_), methanol:WCO molar ratio (*X*_2_), catalyst concentration (*X*_3_), and reaction time (*X*_4_). Specifically, Figure 10a–c display the response surfaces corresponding to the interaction of variable *X*_1_ with variables *X*_2_, *X*_3_, and *X*_4_, respectively. Based on the analysis of these figures, it is observed that the *Y*_EC_ value increases significantly with increasing *X*_1_, which aligns with temperature being the most predominant variable in the process (73.4% contribution). This behavior is attributed to enhanced molecular collision, improved miscibility, and increased mass transfer at higher temperatures, ultimately leading to higher biodiesel ester content.^28,44^

**Figure 10:**
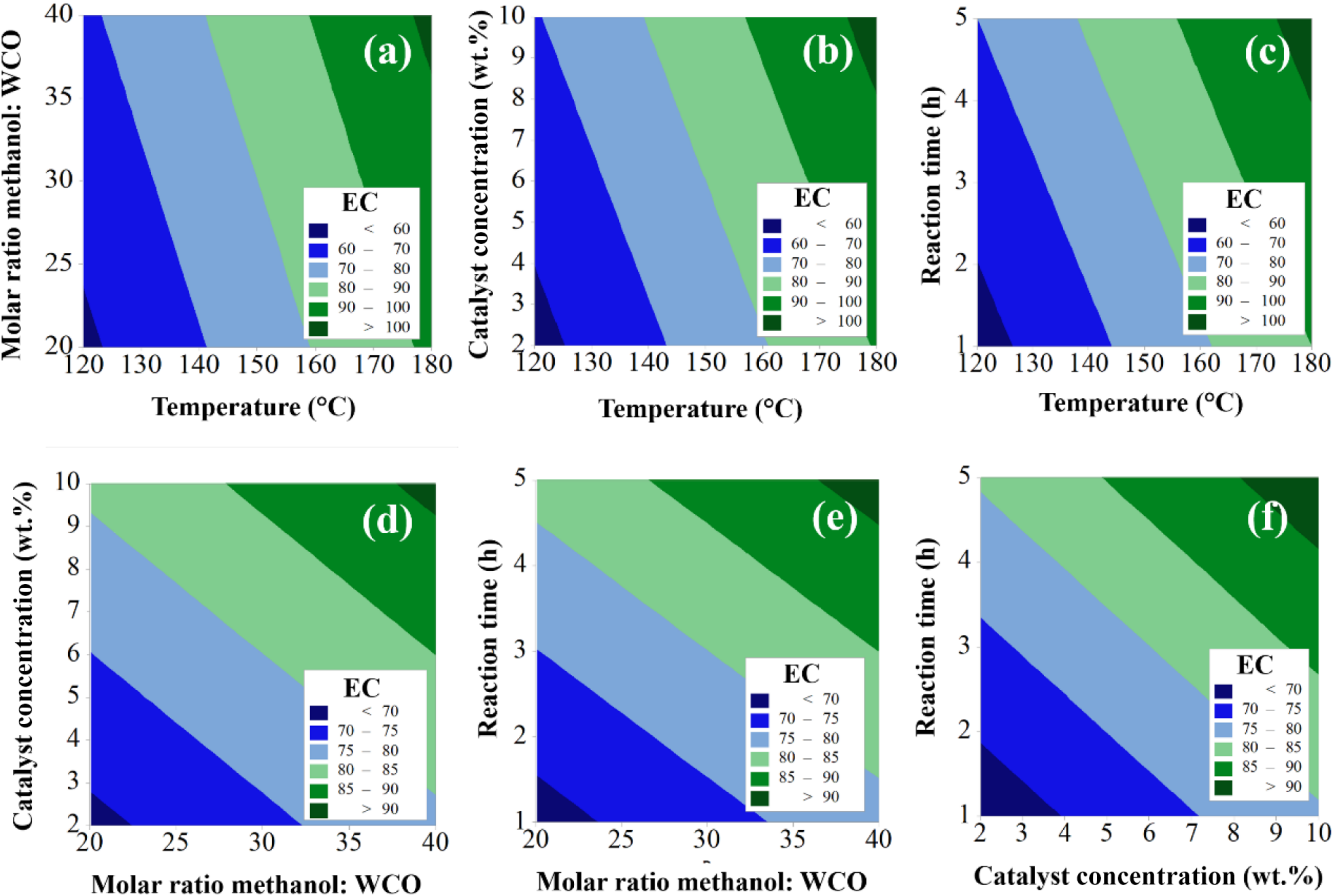
Contour plot of the combined (a) temperature vs. molar ratio methanol:WCO, (b) catalyst amount vs. temperature, (c) temperature vs. reaction time, (d) molar ratio methanol:WCO vs. catalyst amount, (e) reaction time vs. molar ratio methanol:WCO and (f) catalyst amount vs. reaction time.

Analysis of Figure 10d–f indicates that *Y*_EC_ also increases with higher values of *X*_2_, *X*_3_, and *X*_4_. The increase in molar ratio provides greater opportunity for reactant molecular collisions, while higher catalyst concentration elevates the number of active sites available at the solid–liquid interface. Similarly, sufficient reaction time allows the system to approach equilibrium, ensuring complete utilization of active catalytic sites.^17,29^ These factors collectively enhance the simultaneous reaction of triglyceride and alcohol molecules, increasing the overall reaction rate. Furthermore, the acid sites of the catalyst reduce the activation energy of the process, and their greater availability improves the probability of effective collisions, resulting in higher triglyceride conversion.^17,29^

However, although the variables exhibit a directly proportional relationship with *Y*_EC_ values, their optimized values do not necessarily correspond to the maximum values within the studied intervals. This occurs because the independent variables contribute differently to the biodiesel production process, as shown in Table 3. Consequently, the most influential variables (*X*_1_ and *X*_4_) tend to have their optimized values near the upper limits of the studied ranges, while the less predominant variables (*X*_2_ and *X*_3_) tend to provide higher *Y*_EC_ values across broader intervals. This behavior can be observed in the wider contour regions corresponding to high ester content values, particularly in interactions between the lower-impact variables (*X*_2_ and *X*_3_) and the higher-impact variables (*X*_1_ and *X*_4_). The broadening of high-*Y*_EC_ regions indicates that the less influential variables can maintain elevated response values over wider ranges (20:1–40:1 and 2–10 wt %), whereas the more influential variables require more specific conditions within narrower intervals (120–180 °C and 1–5 h) to achieve optimal results.

#### 3.3.1 Model Validation

The optimization of variables *X*_1_ (temperature), *X*_2_ (methanol:WCO molar ratio), *X*_3_ (catalyst concentration), and *X*_4_ (reaction time) for biodiesel synthesis using the 40-MoO_3_/BaFe_2_O_4_ catalyst was successfully achieved. The optimal reaction conditions yielding the highest ester content (*Y*_EC_) were: *X*_1_ = 172 °C, *X*_2_ = 28:1, *X*_3_ = 7.5 wt %, and *X*_4_ = 4 h. Under these conditions, the model predicted a *Y*_EC_ value of 97.1%, demonstrating the efficiency of the optimized process. Furthermore, to evaluate the robustness of the developed model, reactions were conducted at the optimal condition (OC), as well as under two additional random conditions designated R1 (175 °C, 35:1, 4.5 wt %, and 1.5 h) and R2 (145 °C, 25:1, 3.0 wt %, and 2.5 h), as summarized in Table 5.

**Table 5:** Validation Results of the Developed Model

	Reactional conditions	Results (%)		
Exp.	*X*_1_; *X*_2_; *X*_3_; *X*_4_	Observed *Y*_EC_	Predicted *Y*_EC_	Relative error
OC	172; 28:1; 7.5; 4.0	96.4	97.1	0.7
R1	175; 35:1; 4.5; 1.5	85.9	89.7	4.2
R2	145; 25:1; 3.0; 2.5	72.2	69.4	4.0

OC = Optimal condition.

Analysis of Table 5 reveals relative errors of 0.7%, 4.2%, and 4.0% between the model-predicted and experimentally observed values for the OC, R1, and R2 conditions, respectively. These results confirm the predictive efficiency of the developed model within the defined 95% confidence interval.^45^

### 3.4 Examination of Biodiesel Specifications

The properties of biodiesel are crucial for assessing fuel quality relative to established reference standards ASTM D6751 and EN 14214. Accordingly, biodiesel produced using the 40-MoO_3_/BaFe_2_O_4_ catalyst under optimized transesterification conditions (172 °C reaction temperature, 28:1 methanol:WCO molar ratio, 7.5 wt % catalyst concentration, and 4 h reaction time) was evaluated, with results shown in Table 6. The physicochemical properties determined were density at 15 °C, kinematic viscosity at 40 °C, flash point, copper strip corrosion, cold filter plugging point, and acidity, with respective values of 0.873 g cm^–3^, 4.45 mm^2^ s^–1^, 160 °C, 1a, 0 °C, and 0.27 mg KOH g^–1^.

**Table 6:** Features of Biodiesel Produced from WCO Using 40-MoO_3_/BaFe_2_O_4_ Magnetic Catalyst

			Limits		
Properties	Unit	Test methods	ASTM D6751	EN 14214	This study
Density. at 15 °C	g cm^–3^	ASTM D6850	NS	0.86–0.90	0.873
Kinematic viscosity. at 40 °C	mm^2^ s^–1^	ASTM D445	1.9–6.0	3.5–5.0	4.45
Flash point	°C	ASTM D93	>130	>120	160
Copper strip corrosion	–	ASTM D130	<3	1	1a
Cold filter plugging point	°C	ASTM D6371	NS	NS	0.0
Acid value	mg KOH g^–1^	ASTM D664	<0.8	<0.5	0.27

NS = Not specified.

These properties are directly related to vehicle engine performance, fuel supply systems, accumulation of impurities in components, corrosion of metallic engine parts, and fuel temperature sensitivity, particularly important when considering biodiesel use in low-temperature regions.^17,24^ All characterization results suggest a high-quality biofuel that complies with international standards.

### 3.5 Catalyst Recyclability

The magnetic catalyst 40-MoO_3_/BaFe_2_O_4_ was evaluated for recyclability through multiple reaction cycles under optimal transesterification conditions: 172 °C reaction temperature, 28:1 methanol:WCO molar ratio, 7.5 wt % catalyst concentration, and 4 h reaction time, as shown in Figure 11.

**Figure 11:**
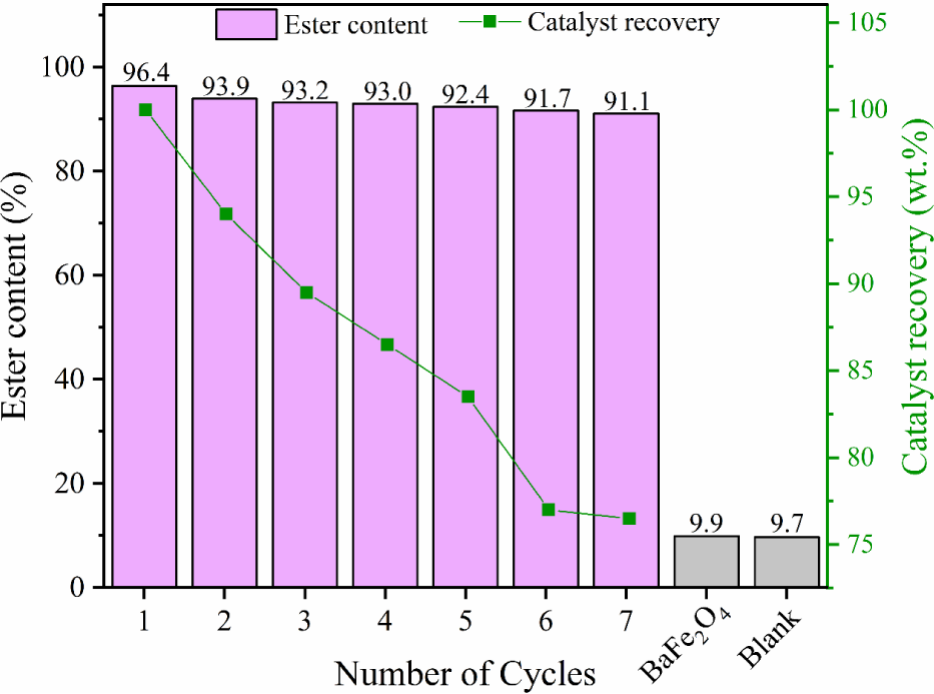
Recyclability of the catalyst 40-MoO_3_/BaFe_2_O_4_.

Analysis of Figure 11 reveals a *Y*_EC_ of 96.4% for the biodiesel produced in the first reaction cycle, with maintained high ester conversion values (*Y*_EC_ > 90%) throughout seven consecutive cycles, indicating exceptional catalytic stability of the studied catalyst. Furthermore, the significant influence of the 40-MoO_3_/BaFe_2_O_4_ catalyst on the process is evident when comparing with control experiments: using only BaFe_2_O_4_ resulted in *Y*_EC_ = 9.9%, while the reaction blank yielded *Y*_EC_ = 9.7%, both substantially lower than values achieved with the developed catalyst.

Catalyst recovery using an external magnetic field proved efficient, with approximately 75% mass recovery after seven reaction cycles. This finding is corroborated by comparing the magnetization curves of fresh and spent catalysts (Figure 12a). Although the saturation magnetization decreased from 19.2 emu g^–1^ to 10.2 emu g^–1^ after seven cycles, the spent catalyst retained sufficient magnetization for effective separation from the reaction medium by magnetic decantation.

**Figure 12:**
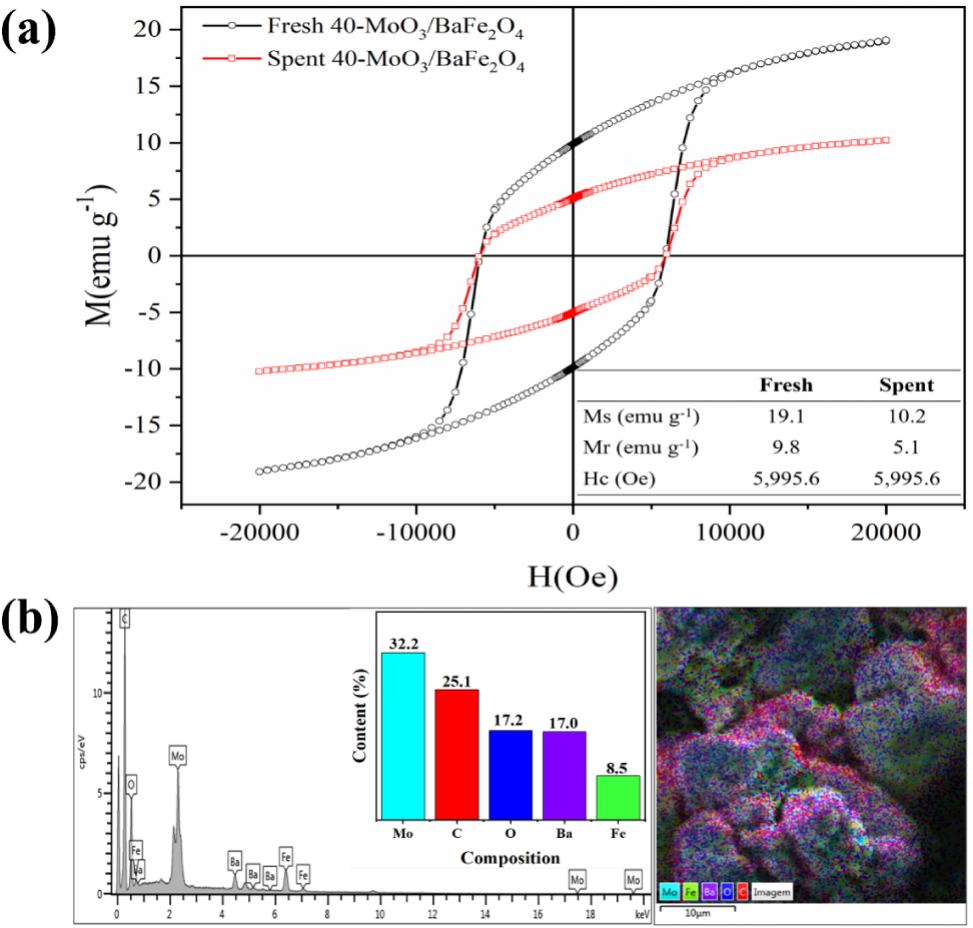
(a) VSM and (b) composition of the spent catalyst 40-MoO_3_/BaFe_2_O_4_.

The decrease in the catalyst’s Ms after seven reaction cycles is probably due to the presence of carbon (nonmagnetic material) from reagents that did not react in the process and were not removed in the washing stage. As a result, the percentage of nonmagnetic material increased and, since VSM analysis is performed in relation to mass, the Ms value decreases, but not to the point of impairing the separation of the catalyst by means of a magnetic field.

In addition, the small decrease in reaction conversion over the reaction cycles can be explained by the leaching that occurred on the catalyst surface (Figure 12b), as there is a decrease in the molybdenum content in the catalyst after the seventh reaction cycle (41.75%) compared to the new catalyst (32.2%). In addition, the presence of carbon (C) at a high percentage of 25% is noted, which contributes to the obstruction of the active sites of the catalyst and the consequent decrease in its catalytic activity.^17,23,24^

### 3.6 Proposed Mechanism Using the 40-MoO_3_/BaFe_2_O_4_Catalyst for Biodiesel Production

A mechanism for the transesterification of WCO catalyzed by 40-MoO_3_/BaFe_2_O_4_ is illustrated in Figure 13. The reaction follows three fundamental stages. In the first step, protonation, the acid sites of the catalyst interact with the carbonyl group of the triglyceride molecules, generating a positively charged carbocation that increases the electrophilicity of the carbonyl carbon and promotes the reaction’s progress.^24,35^ The second step corresponds to the nucleophilic attack, in which methanol acts as a nucleophile, its oxygen atom donates electrons to the carbocation through the methoxide ion (CH_3_O^–^), forming a transient tetrahedral intermediate.^11–13^

**Figure 13:**
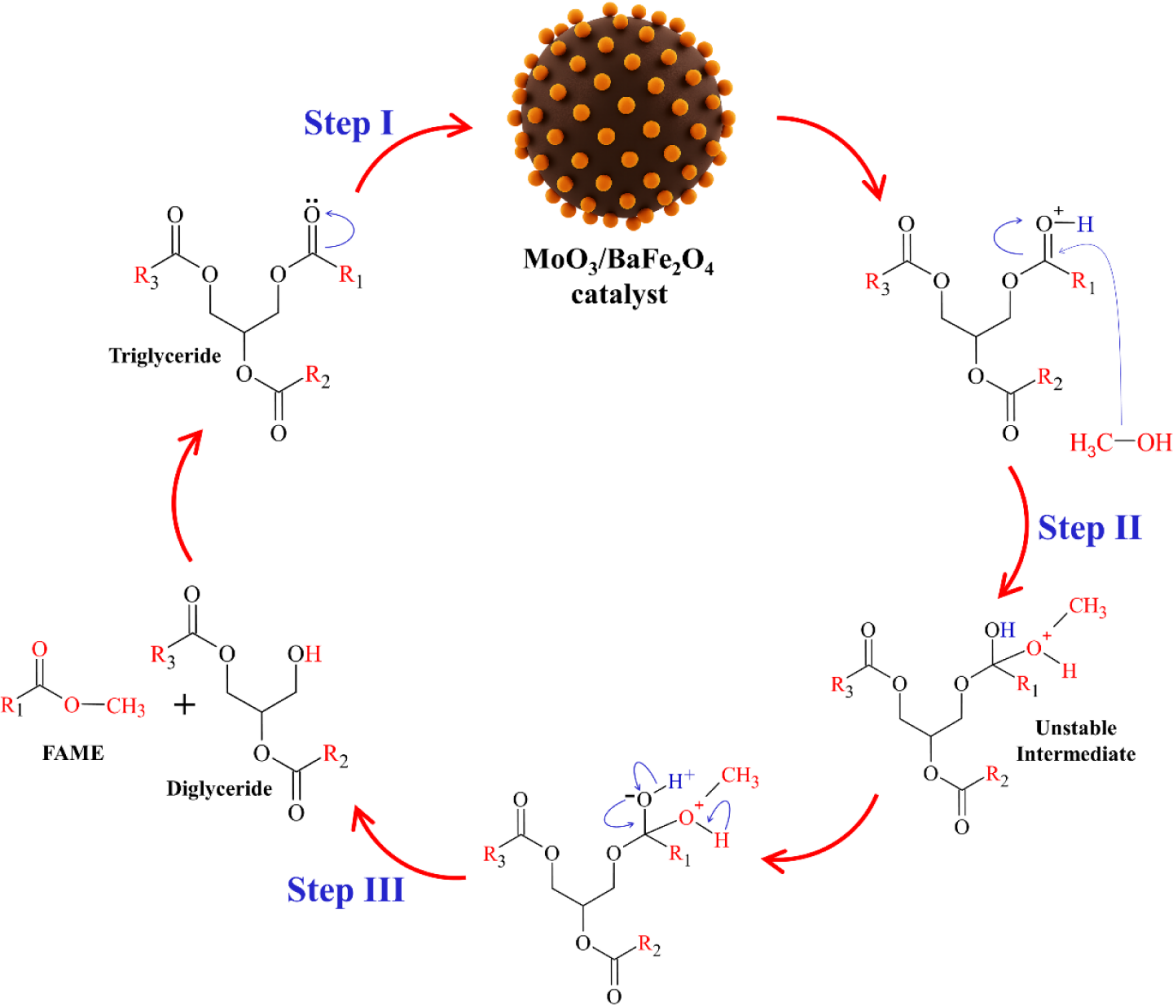
Proposed mechanism using the 40-MoO_3_/BaFe_2_O_4_ catalyst.

The final step, elimination, involves the decomposition of this intermediate, releasing the OHR′ group and producing methyl esters (biodiesel). During this stage, the catalyst is regenerated, maintaining its active sites available for subsequent reaction cycles.^13,14^

### 3.7 Feedstock Assessment

The performance of the 40-MoO_3_/BaFe_2_O_4_ catalyst was analyzed in the transesterification reaction using different types of oils, including refined soybean oil, waste cooking oil (WCO), buriti oil (*Mauritia flexuosa*), palm kernel oil (*Elaeis guineenses*), and andiroba oil (*Carapa guianensis*). Figure 14 shows the behavior of the catalyst in the tests performed with these raw materials and shows that all biodiesels obtained exhibited an ester content greater than 90%. This result demonstrates the versatility of the catalyst in biodiesel production, since it preserves its catalytic efficiency both with refined raw materials, whose acidity values are 0.2 mg KOH g^–1^ (soybean oil) and 1.6 mg KOH g^–1^ (palm kernel oil), as well as with lower quality raw materials, which have higher acidity, 2.2 mg KOH g^–1^ (WCO), 33.7 mg KOH g^–1^ (buriti), and 18.9 mg KOH g^–1^ (andiroba).

**Figure 14:**
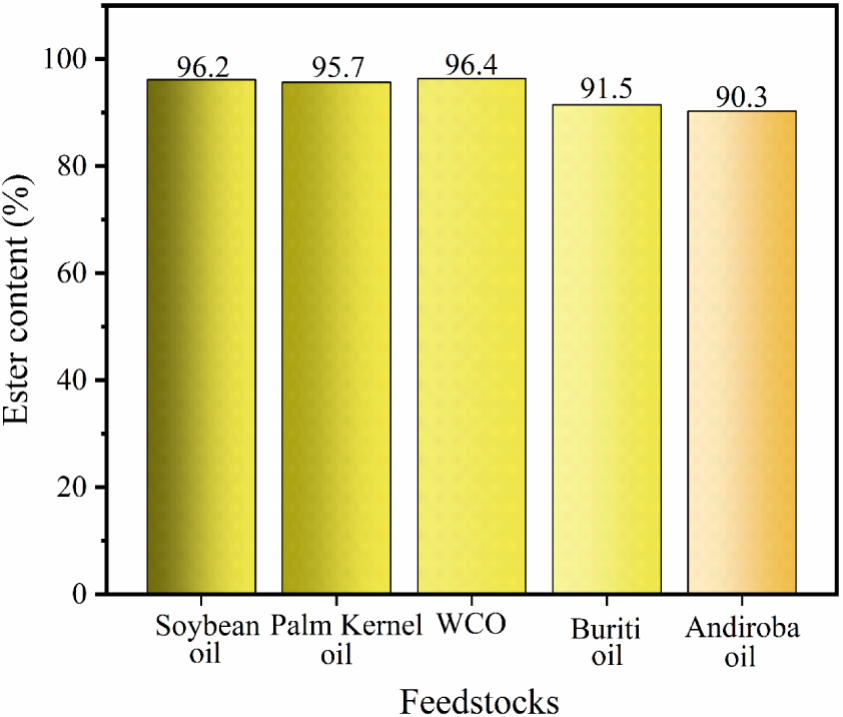
Evaluation of several feedstocks.

### 3.8 Comparative Study of the Performance of Different Acid Catalyst in Transesterification

Table 7 compares different heterogeneous acid catalysts in terms of their respective catalytic activities and reaction parameters used for biodiesel production, including the 40-MoO_3_/BaFe_2_O_4_ catalyst developed in this study. Through comparative analysis, the biodiesel produced using the 40-MoO_3_/BaFe_2_O_4_ catalyst shows a significant *Y*_EC_ value that is comparable to those reported in the literature. Furthermore, the main advantage of using the magnetic catalyst developed in this work is its catalytic activity and stability under milder operational conditions, combined with the possibility of faster and more efficient magnetic separation, a feature not offered by conventional catalysts, which require more costly separation methods such as filtration and centrifugation.

**Table 7:** Comparing the Stability of the Catalyst Synthesized in This Study with Previous Works

		Reaction conditions					
Catalyst	Feedstocks	*X*_1_	*X*_2_	*X*_3_	*X*_4_	*Y*_EC_ (%)	References
MoO_3_/SrFe_2_O_4_	WCO	164	40	10	4	95.4	Gonçalves et al. (2021)^14^
HPMo/TiO_2_	WCO	190.0	90:1	5.0	4.0	94.5	Gonçalves et al. (2021)^15^
WO_3_/CuFe_2_O_4_	WCO	180.0	45:1	6.0	3.0	95.2	Santos et al. (2022)^24^
Nb_2_O_5_/SO_4_	Macaw oil	250.0	120:1	30.0	4.0	99.2	Conceição et al., (2016)^46^
Fe_2_O_3_–MnO–SO_4_/ZrO_2_	WCO	180.0	20:1	3.0	6.0	96.0	Alhassan et al., (2015)^47^
HPMo/Nb_2_O_5_	Macaw oil	210	90	20	4	99.6	Conceição et al., (2017)^48^
40-MoO_3_/BaFe_2_O_4_	WCO	172	28	7.5	4.0	96.4	**Present study**

## 4 Conclusions

The acidic magnetic catalyst 40-MoO_3_/BaFe_2_O_4_ was successfully synthesized and applied in the sustainable production of biodiesel from WCO. Under Taguchi L_9_-optimized conditions (172 °C, methanol:WCO 28:1, 7.5 wt % catalyst, 4 h), a high ester conversion of 96.4% was achieved, supported by a robust predictive model (*R* ^2^ = 0.9410) with errors below 5% between predicted and experimental values. The catalyst exhibited exceptional recyclability, maintaining >90% conversion over seven cycles due to its magnetic separability and structural stability, outperforming conventional catalysts by enabling efficient recovery without costly separation methods. These results demonstrate the dual advantage of 40-MoO_3_/BaFe_2_O_4_, such as high catalytic efficiency under moderate conditions and sustainable operation through magnetic recovery, positioning it as a promising candidate for scalable biodiesel production from low-cost feedstocks such as WCO.

## Supplementary Material



## Notes

Methodologies for determining the physicochemical properties of WCO: Acid Value (AOCS Cd 3d–63); Saponification value (AOCS Tl 1a-64); Viscosidade cinemática a 40 °C (ASTM D445); Moisture content (AOCS Ca 2b–38) (PDF)
