# Supplementary material for: A New Magnetically Separable BaFe2O4 Acid Catalyst for Sustainable Biodiesel Production: L9 Taguchi Optimization and Robust Recyclability
Source: ACS Omega. 2026 Jan 27;11(5):8046–60. doi: 10.1021/acsomega.5c10408 (PMC12902983; doi:10.1021/acsomega.5c10408)
Supplement: Supplementary file 1 [file ao5c10408_si_001.pdf]

Supplementary material

A new magnetically separable $BaFe_2O_4$ acid catalyst for sustainable biodiesel production: L_9 Taguchi optimization and robust recyclability

Matheus Arrais Gonçalves[a], Hiarla Cristina Lima dos Santos[a], Vicente da Silva Lima[b], Heverton Jonnys Feitosa da Silva[a], Deborah da Cunha Fonseca[c], Thaissa Saraiva Ribeiro[a], Beatriz dos Santos Silva[a], Alexandre da Cas Viegas[d], Leyvison Rafael Vieira da Conceição[a,c*]

[a] Federal University of Pará, Institute of Exact and Natural Sciences, Graduate in Chemistry Program, Laboratory of Catalysis and Oleochemical, 66075–110, Belém, Pará, Brazil.

[b] Federal University of Pará, Institute of Biological Sciences, Graduate in Biotechnology Program, 66075–110, Belém, Pará, Brazil.

[c] Federal Institute of Education, Science and Technology of Pará, Graduate Program in Materials Engineering, 66093–020, Belém, Pará, Brazil.

[d] Federal University of Rio Grande do Sul, Institute of Physics, 90035–190, Porto Alegre, Rio Grande do Sul, Brazil.

*Corresponding author

E-mail address: rafaelvieira@ufpa.br (Conceição, L.R.V)

Methodology for determining the physicochemical properties of WCO

Acid Value (AOCS Cd 3d–63): A 10 g oil sample was weighed into a 250 mL Erlenmeyer flask and dissolved in 125 mL of a neutralized isopropyl alcohol:toluene mixture (1:1 v/v). The solution was titrated with a standardized 0.1 M ethanolic potassium hydroxide (KOH) solution using phenolphthalein (1% w/v) as an indicator. The endpoint was identified by the first appearance of a persistent pale-pink color that lasted for at least 30 seconds. A blank titration was performed concurrently. The acid value, expressed as mg of KOH per gram of sample (mg KOH g^{-1}), was calculated using the following equation:

$$\text{Acid Value} = \frac{(V_S - V_b) . C . 56{,}1}{m}$$

where:

- V_s is the volume of KOH consumed by the sample (mL),
- V_b is the volume of KOH consumed by the blank (mL),
- C is the concentration of the KOH solution (mol L^{-1}),
- m is the mass of the sample (g).

Saponification value (AOCS Tl 1a-64): Approximately 2 g of the oil sample was weighed into a round-bottom flask, followed by the addition of 25.0 mL of a 0.5 N alcoholic potassium hydroxide (KOH) solution. The mixture was refluxed for 60 min with agitation. After cooling, the solution was titrated with a standardized 0.5 N hydrochloric acid (HCl) solution using phenolphthalein (1%) as an indicator. A blank assay was performed following the identical procedure. The saponification matter content, expressed as mg KOH per gram of sample (mg KOH g^{-1}), was calculated using the equation:

$$\text{Saponification value} = \frac{(V_b - V_s) . C . 56{,}1}{m}$$

where:

- V_b is the volume of HCl consumed by the blank (mL),
- V_s is the volume of HCl consumed by the sample (mL),
- C is the concentration of the HCl solution (mol L^{-1}),

- m is the mass of the sample (g).

Viscosidade cinemática a 40 °C (ASTM D445): The measurement was performed using a Cannon-Fenske viscometer (SCHOTT GERÄTE, model 520 23) immersed in a thermostatic bath to maintain a constant temperature of 40.00 ± 0.05 °C. The viscometer was loaded with the oil sample, and after a thermal equilibration period, the flow time between the calibration marks was measured with a digital stopwatch. The procedure was carried out in triplicate. The kinematic viscosity (ν), expressed in square millimeters per second ($mm^2\ s^{-1}$), was calculated from the average flow time (t) and the viscometer constant (C) using the equation:

$$\text{Viscosity} = \text{C}\,.\,\text{t}$$

where:

- C is the viscometer constant ($mm^2\ s^{-2}$),
- t is the average flow time (s).

Moisture content (AOCS Ca 2b–38): Porcelain dishes were initially cleaned and dried in an oven at 105 °C for 1 h, then conditioned in a desiccator until completely cooled for taring. Approximately 10 g of the oil sample was weighed into each tared dish. The dishes containing the sample were then placed in an oven at 105 ± 1 °C for 1 h. After this period, the dishes were transferred to a desiccator, cooled to ambient temperature, and weighed again. This cycle of heating, cooling, and weighing was repeated until the mass difference between two successive weighings was less than 0.1%. The moisture content (U), expressed as a percentage (%), was calculated using the equation:

$$\text{Moisture content (\%)} = \frac{(m_1 - m_2)}{m_a}.100$$

where:

- m_1 is the mass of the dish and sample before drying (g),
- m_2 is the mass of the dish and sample after drying (g),

- m_a is the initial mass of the sample (g).
